# A Broad Spectrum of Liver Manifestations in Common Variable Immunodeficiency Syndrome—Two Case Reports and a Literature Overview

**DOI:** 10.3390/diagnostics15131659

**Published:** 2025-06-29

**Authors:** Eva Supovec, Jan Drnovšek

**Affiliations:** 1 Department of Gastroenterology, University Medical Centre Ljubljana, 1000 Ljubljana, Slovenia; 2 Faculty of Medicine, University of Ljubljana, 1000 Ljubljana, Slovenia

**Keywords:** CVID, common variable immunodeficiency, liver involvement, nodular regenerative hyperplasia, non-cirrhotic portal hypertension, autoimmune hepatitis, granulomatous hepatitis, enteropathy, gut–liver axis, hepatic cancer

## Abstract

**Background and Clinical Significance:** Common variable immunodeficiency (CVID) is a primary B-cell immunodeficiency disorder, characterized by severe hypogammaglobulinemia and disturbed antibody production. In addition to increased susceptibility to recurrent respiratory and gastrointestinal infections, CVID can lead to a wide array of complications associated with immune dysregulation, which can also affect the liver. Liver involvement occurs in about 10% of patients with CVID, and can result from a range of causes, including infections, autoimmune disorders, lymphoproliferative conditions, granulomatous inflammation, and infiltrative processes. The most common liver manifestations include nodular regenerative hyperplasia, granulomatous or autoimmune hepatitis, and lymphocytic infiltration. The prevalence, pathophysiology, extent, and prognosis of liver involvement in CVID have not been systematically studied. **Case Presentation:** The object of this article is to present two patients with CVID-related liver disease and to illuminate the most relevant causes of liver involvement in CVID, describe the clinical features of their liver disease, and summarize the diagnostic and therapeutic approaches for its management. **Conclusions:** Liver involvement is an expected complication in patients with CVID syndrome. The delayed recognition of this pathology significantly worsens the disease prognosis, making the early detection of this potential complication crucial.

## 1. Introduction

Common variable immunodeficiency (CVID) is the most frequent symptomatic antibody deficiency diagnosed in adulthood, with an estimated prevalence of 1/10,000 to 1/100,000. CVID is characterized by hypogammaglobulinemia (low levels of IgG and either IgA or IgM isotypes) and impaired antibody production, which are a result of the dysfunctional maturation of B-cells [1,2,3]. Based on the International Consensus Document (ICON) guidelines, it is defined by four main criteria: (1) a low IgG level (below the age-appropriate range on two tests at least 3 weeks apart, unless very low—typically <100–300 mg/dL depending on age), (2) low IgA and/or IgM levels, (3) poor antibody responses to vaccination, and (4) the exclusion of other causes of hypogammaglobulinemia [1]. Rather than a single disease, CVID encompasses a range of genetic [4,5,6,7,8,9,10,11,12] and immunologic disorders [13,14], as patients may also present with T-cell or antigen-presenting cell abnormalities [14,15,16]. The median age of onset is between the ages of 20 and 40 [16,17,18,19,20]. CVID can present with a diverse clinical picture, affecting various organ systems. The hallmarks include recurrent infections of the respiratory and gastrointestinal tracts, as well as numerous disorders associated with immune dysregulation, such as autoimmunity, granulomatosis, lymphoproliferation, and malignancies, all of which can also affect the liver [15,21,22,23,24]. Liver involvement in CVID has not been well described, as there is limited evidence about its prevalence, pathogenesis, clinical manifestations, management, and treatment [16,25]. It can present with various histopathological patterns, such as nodular regenerative hyperplasia, autoimmune or viral hepatitis, inflammatory granulomas, fibrosis, cirrhosis, etc. Liver involvement in CVID is often overlooked due to its insidious course. A delayed diagnosis can lead to severe liver damage and a poor prognosis [15,26,27]. In this case report with a literature overview, we present two CVID patients who were admitted to our department due to clinical findings of liver dysfunction and abnormal liver tests, respectively. We review the current literature regarding manifestations of liver disease in CVID.

## 2. Case Reports

### 2.1. Patient 1

**Figure 1:**
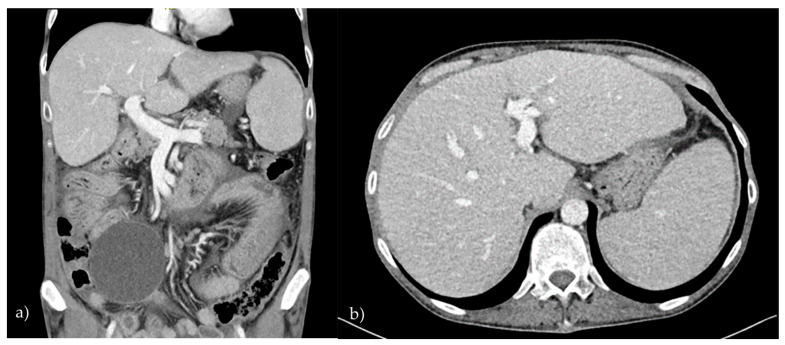
**Computer tomography—(a) sagittal view, (b) coronal view:** Hepatosplenomegaly, the dilation of the portal vein in the hepatic hilum, the presence of free fluid (ascites) and localized fluid collections in the abdominal cavity, and edema of the mesenteric fat.

A 46-year-old male with CVID was admitted to our department with symptoms of ascites and elevated liver enzymes. Over the preceding three months, the patient had experienced persistent, moderate-intensity abdominal pain along with progressive abdominal distension. He also reported generalized fatigue and anorexia. On physical examination, his abdomen was distended, tense, and diffusely tender to palpation, with evident ascites. The remainder of the physical examination was unremarkable. The laboratory investigations revealed elevated levels of alkaline phosphatase (AP) (247.31 U/L; reference range: 43–115 U/L) and gamma-glutamyl transferase (GGT) (70.66 U/L; reference range: <55 U/L). A contrast-enhanced computed tomography (CT) of the abdomen demonstrated hepatosplenomegaly, significant ascites, and pronounced inflammation in both the stomach and duodenal wall (Figure 1). An esophagoduodenoscopy with tissue sampling revealed inflammation of the stomach and villous atrophy of the duodenum, resembling the pattern seen in celiac disease; however, there were insufficient intraepithelial lymphocytes to fulfill the diagnostic criteria for celiac disease. A helicobacter pylori infection was ruled out. A paracentesis of the ascites was performed, and the biochemical analysis indicated transudative fluid, which was sterile on the microbiological examination. A liver biopsy was subsequently carried out, and the histopathological evaluation revealed dilated portal tracts with focal proliferation of the connective tissue and inflammatory infiltrates. Based on these findings, a diagnosis of non-cirrhotic portal hypertension secondary to CVID was established. The patient’s ascites was effectively managed with diuretic therapy.

### 2.2. Patient 2

A 37-year-old female with a known diagnosis of CVID presented to our outpatient clinic due to abnormal liver function. The laboratory findings revealed elevated AP at 721.56 U/L (reference range: 33–98 U/L), G-GT at 409.58 U/L (reference range < 38 U/L), aspartate transaminase (AST) at 92.77 U/L (reference range < 32 U/L), and alanine transaminase (ALT) at 92.77 U/L (reference range: <35 U/L). The patient had previously been managed by a gastroenterologist for suspected malabsorption, a condition associated with CVID. Her medical history was also significant for adrenal insufficiency, secondary diabetes, and bronchiectasis. Additionally, she had undergone a splenectomy for a significant splenomegaly, with the histopathological findings consistent with T-cell hyperplasia characteristic of CVID. At the time of presentation, the patient was clinically asymptomatic. A comprehensive number of laboratory tests were conducted to investigate the cause of the hepatopathy, including serological assays for viral hepatitis and celiac disease; a review of the proteinogram; and measurements of ceruloplasmin, serum copper, alpha-1 antitrypsin, and HEP-2 autoantibodies. However, no definitive etiology was identified. The imaging studies, including an abdominal ultrasound and CT, revealed a mildly enlarged liver with a homogenous parenchymal architecture. A liver biopsy was performed to further elucidate the cause of the liver dysfunction. The histopathological examination revealed changes suggestive of autoimmune hepatitis, with moderate portal inflammation, interface hepatitis, and advanced fibrosis. After review by a multidisciplinary team, a dual immunosuppressive regimen was initiated. However, the therapy failed to improve the liver enzyme levels and was discontinued due to adverse effects, including hair loss and gastrointestinal discomfort. During the subsequent follow-ups, a persistent elevation in the liver enzyme levels was observed, with the AP increasing to 1043.11 U/L (reference range: 33–98 U/L), the AST to 228.74 U/L (reference range: <32 U/L), the ALT to 159.88 U/L (reference range: <35 U/L), and the gamma-GT to 761.08 U/L (reference range: <38 U/L). A repeat abdominal ultrasound revealed substantial hepatomegaly. A second liver biopsy demonstrated a worsening of the pre-existing condition, including the impairment of at least 50% of the liver parenchyma, intense lobular inflammation, and non-suppurative granulomas of the sarcoid type, consistent with the diagnosis of granulomatous hepatitis. Systemic glucocorticoid therapy was initiated, after which the liver function test levels decreased slightly, but had still not reached reference ranges.

## 3. Discussion with Literature Overview

The prevalence of liver disease in patients with CVID varies across studies, largely due to differences in the diagnostic criteria applied [22]. A recent meta-analysis estimated the prevalence to range between 9% and 79% [23]. The clinical presentation of liver involvement is highly variable, spanning from mildly elevated liver enzymes to more severe conditions, such as hepatic decompensation and liver failure [15]. The signs and symptoms can often be subtle, with an insidious and unexpected onset. Some patients may remain asymptomatic, presenting only with abnormal liver function tests or imaging findings, such as hepatomegaly or splenomegaly on an abdominal ultrasound. Others may report nonspecific symptoms, including fatigue, nausea, vomiting, pruritus, and abdominal pain. On clinical examination, liver disease can manifest with jaundice, ascites, peripheral edema, hepatomegaly, splenomegaly, esophageal varices, or other signs indicative of portal hypertension or cirrhosis [15,28].

Liver involvement in CVID can result from various etiologies, including infections (such as viral hepatitis or an extra-intestinal localization of Giardia lamblia), immune dysregulation (e.g., nodular regenerative hyperplasia, granulomas, or lymphocytic infiltration), or malignancies (such as primary liver cancer, metastatic gastrointestinal adenocarcinomas, or lymphomas) [23]. Additionally, liver disease in CVID may be linked to a dysregulation of the gut–liver axis [15].

Nodular regenerative hyperplasia (NRH) is the most common liver lesion observed in patients with CVID [1,16,28,29,30]. Histologically, NRH is characterized by parenchymal nodules without accompanying fibrosis or cirrhosis [31]. It is believed to result from intrahepatic vasculopathy, which impairs blood flow and subsequently leads to hepatocyte injury and nodular regeneration [16,30]. While the exact etiology of NRH remains largely unknown, three primary hypotheses have been proposed [22]. The first suggests that thrombosis of the small portal vein branches obstructs microvascular perfusion [30,32]. The second implicates immune dysregulation, where chronic lymphocytic infiltration of the liver, mediated by CD8+ lymphocytes, causes portal vein endothelitis [33,34]. The third hypothesis points to a dysfunction of the gut–liver axis, with increased intestinal permeability enabling the microbial translocation of intestinal bacteria and endotoxins into portal circulation [23,35], thereby inducing liver inflammation [22,23,25,36]. The diagnosis of NRH can be challenging due to its often asymptomatic nature, as it may initially present only with elevated AP and GGT levels [16,23,30]. This was likely the case with our first patient, who had probably been asymptomatic for some time and was diagnosed only after clear signs of portal hypertension had developed. Moreover, the detection of NRH is complicated by the fact that regenerating nodules are typically too small or isechogenic to be identified through abdominal ultrasound [37].

While NRH was previously considered to follow a relatively benign course, it is now recognized that it frequently progresses to non-cirrhotic portal hypertension (NCPH) [15,29,30] as the regenerative nodules compress the portal and central veins [30,31,38], thus significantly altering the blood flow through the portal system. The primary clinical manifestation of NCPH is splenomegaly, often accompanied by hypersplenism, neutropenia, and thrombocytopenia, further compromising the ability of CVID patients to manage infections [30]. Other clinical features of NCPH include ascites, esophageal and gastric varices, and hepatomegaly [28,29,30,33]. Notably, symptoms commonly associated with cirrhosis, such as gynecomastia, palmar erythema, and spider angiomas, are typically absent in NCPH [37]. Although both of our patients presented with hepatomegaly and splenomegaly, the first patient exhibited ascites due to NCPH; on the other hand, in the second patient, their splenomegaly was not attributed to NCPH but rather to immune dysregulation, as lymphocytic infiltration of various organs is a common feature of CVID [19,22,24].

As in our second patient, a proportion of CVID patients with NRH either develop or initially present with autoimmune hepatitis (AIH). It is likely that AIH represents a severe stage of NRH, as AIH’s pathohistological features (interface hepatitis, prominent bridging periportal and perisinusoidal fibrosis, lymphocytic infiltration, etc.) can be superimposed onto the underlying parenchymal nodules indicative of NRH. AIH evolves more rapidly, with signs of impaired liver function and portal hypertension appearing earlier and usually results in severe liver dysfunction as treatment is often ineffective, as we observed in our second patient [30].

Furthermore, our second patient later exhibited features of granulomatous hepatitis. While granulomatous disease in CVID predominantly affects the lungs, lymph nodes, and spleen [1,26,39,40], inflammatory granulomatous lesions in the liver are also commonly observed [29].

The granulomas associated with CVID are non-caseating and not linked to a mycobacterial infection [29]. The precise cause of granuloma formation remains unclear, but has been associated with dysregulated macrophage activation and T-cell deficiencies [13,41]. These granulomas can sometimes be mistaken for sarcoidosis, particularly if they are detected before a diagnosis of CVID is established. Differentiation between the two conditions can be established by measuring the serum immunoglobulin levels, which are typically normal or elevated in sarcoidosis but decreased in CVID. The main liver manifestations of CVID are summarized in Table 1.

As previously discussed, liver injury can result from microbial translocation due to increased intestinal permeability, a condition commonly associated with chronic small bowel enteropathy in CVID. This non-infectious gastrointestinal disorder, which can mimic celiac, affects approximately 10% of CVID patients [42]. While the underlying mechanism remains unclear, it is likely autoimmune in nature, stemming from immune dysregulation [43]. The most frequent symptom is persistent chronic diarrhea [43,44], which can lead to weight loss and malnutrition due to severe malabsorption and steatorrhea, resulting in a loss of proteins, minerals, and fat-soluble vitamins [42,44,45]. The histopathological findings are similar to those in celiac disease, with villous atrophy and intraepithelial lymphocytosis being the most common features [42,44]. The distinctive characteristics differentiating the two diagnoses include the severe depletion of intestinal plasma cells and follicular lymphoid hyperplasia, which are only seen in CVID enteropathy [42,44,46]. Celiac-specific antibodies are typically absent, and most patients do not possess the HLA genes associated with celiac disease, nor do they respond to a gluten-free diet [42,43,44,46]. A diagnosis of CVID enteropathy is likely in both of our patients. The first patient presented with the classic histopathological signs of enteropathy, while the second patient displayed the clinical features of malabsorption. The latter may benefit from an endoscopic biopsy to obtain a definitive histopathological diagnosis. In cases of CVID enteropathy, treatment with immunosuppressive corticosteroids is recommended, as the standard CVID therapy involving regular intravenous immunoglobulin (IVIg) replacement does not typically alleviate symptoms or reduce intestinal inflammation [42,44]. In addition, watchful follow-up is recommended, as epithelial exudation can occur, leading to the further depletion of the immunoglobulin with an increased susceptibility to infections and increased requirements for parental immunoglobulin supplementation [42].

The first patient also presented with chronic, although H. pylori-negative, gastritis upon a gastroscopy. This condition predisposes the patient to gastric cancer, which, along with non-Hodgkin lymphoma (NHL) and non-melanoma skin cancer (NMSC), is among the most prevalent malignancies in individuals with CVID [20,26,42,47,48,49,50]. Other established risk factors for gastric cancer include H. pylori infection, recurrent gastrointestinal infections, and atrophic gastritis [47]. The overall prevalence of malignancy in CVID is estimated to be approximately 10% [47,49]. The genetic mutations associated with primary immunodeficiency disorders may either directly elevate the risk of cancer or indirectly facilitate carcinogenesis by promoting genetic instability, sustained lymphoproliferation, and/or oncogenic infections [51,52]. Furthermore, manifestations of immune dysregulation, such as arthritis, atrophic gastritis, and interstitial lung disease, have been identified as additional risk factors for malignancy in CVID [47]. Malignant neoplasms in CVID tend to occur at a younger age, with an average onset of 45 years, compared to the general population [47,53]. This phenomenon contributes significantly to the increased mortality observed in these patients. Interestingly, despite the high prevalence of liver disease in CVID, there has been no observed increase in the incidence of hepatic cancers. To date, only two cases of HCC in individuals with CVID have been reported. The first case involved a 15-year-old girl with CVID and hepatitis C, who developed end-state hepatocellular carcinoma (HCC) over a period of four years [54]. The second concerned a 50-year-old male who died within 3–4 weeks due to rapidly progressive HCC in the absence of any pre-existing liver disease [55]. The relative rarity of hepatic malignancies in CVID patients could possibly be explained by their reduced life expectancy, which limits the likelihood of reaching the advanced age at which chronic hepatitis—whether viral, autoimmune, or otherwise—would typically predispose individuals to the development of HCC.

Other potential causes of liver abnormalities in CVID include gall bladder disease with cholestasis, primary biliary cirrhosis, primary sclerosing cholangitis, liver cirrhosis, and viral hepatitis [1,56,57]. A liver biopsy plays a crucial role in the diagnostic work-up of liver abnormalities, providing histopathological confirmation and distinguishing between overlapping conditions. In CVID patients, a percutaneous liver biopsy may be complicated by thrombocytopenia and coagulation disorders, increasing the risk of bleeding. Thrombocytopenia may result from CVID (e.g., immune thrombocytopenia [58]) or from advanced liver disease. As such, a biopsy is typically reserved for patients without portal hypertension and with platelet counts above 50 × 10^9^/L [59]. In cases with severe thrombocytopenia, alternative approaches should be considered.

Historically, intravenous immunoglobulin (IVIg) therapy has been a significant cause of iatrogenic viral hepatitis—such as hepatitis B (HBV), hepatitis C (HCV), cytomegalovirus (CMV), and Epstein–Barr virus (EBV)—in individuals with CVID [60,61,62]. However, advancements in the viral screening of donated plasma have markedly reduced the incidence of such infections, making them exceedingly rare unless additional risk factors are present. Despite these improvements, when hepatitis is identified from a liver biopsy, it remains essential to exclude chronic viral infections through comprehensive serological testing and viral load measurements, as specific antibodies (e.g., anti-HCV antibodies) may yield false-negative results due to the underlying immunodeficiency [15,25].

There is currently no standardized diagnostic approach for liver disease in patients with CVID [16]. Nevertheless, a proposed diagnostic workup includes a combination of laboratory and imaging studies to comprehensively assess liver function. These may include a complete blood count, liver function tests, coagulation profile, and serological and viral load assessments for hepatitis B (HBV) and hepatitis C (HCV). Imaging modalities, such as abdominal ultrasound, portal Doppler ultrasound, transient elastography, CT, or magnetic resonance imaging (MRI) of the abdomen, can be employed to assess liver function, estimate the degree of liver fibrosis, detect structural changes in the liver, and estimate splenomegaly and hepatomegaly. Autoimmune hepatitis, like other autoimmune conditions in CVID, presents significant diagnostic challenges. Serological markers, which are commonly used in the diagnosis of autoimmune diseases, are often unreliable in these patients due to low or undetectable levels of specific autoantibodies. Consequently, in cases where AIH, NRH, or liver involvement of unknown etiology is suspected, a liver biopsy becomes essential for establishing a definitive diagnosis [15,16,23,63].

Patients with CVID who develop liver involvement require prompt and aggressive therapeutic intervention. In cases of NRH, AIH, or granulomatous hepatitis, treatment typically includes the use of corticosteroids and immunomodulatory agents, such as azathioprine, 6-thioguanine, and 6-mercaptopurine [15,22]. Although these therapies carry an increased risk of infection, they remain essential for managing these conditions. When NRH arises from a prothrombotic state, anticoagulation therapy is indicated [64]. For patients with portal hypertension, the standard treatment may include the administration of non-selective beta-blockers, such as propranolol or carvedilol; the placement of a transjugular intrahepatic portosystemic shunt (TIPS); and endoscopic ligation of variceal bleeding [16,22,64,65,66,67]. In cases of cirrhosis or end-stage liver disease, a liver transplantation remains the only viable therapeutic option, offering improvements in both survival and quality of life [15,16,23,68]. However, a liver transplantation in CVID patients is approached with risks from lifelong immunosuppression and severe infections. To date, only approximately 30 cases of liver transplantation in CVID patients have been documented in the literature [69,70,71,72].

Given the complexity of liver disease in CVID, its effective management requires close collaboration between immunologists and hepatologists. Immunologists focus on controlling immune dysfunction and the minimization of infection risk, while hepatologists manage the liver-specific complications and guide the therapeutic interventions, ensuring comprehensive care. However, despite the implementation of appropriate treatments for liver complications, therapy is often ineffective, and patients die due to progressive liver disease or systemic infection [30]. Patients with CVID generally live into middle age, with a large cohort study reporting a median age at death of 44 years for females (range 10–90) and 42 years for males (range 9–79) [26]. However, liver involvement significantly worsens the prognosis, as patients with liver disease are about seven times more likely to die than those without it [16]. Therefore, patients must be carefully monitored. Blood tests with a liver profile should be repeated every 2–6 months and ultrasound examinations with transient elastography every 12 months to assess the progression of liver damage and to estimate the degree of liver fibrosis [16,23].

This overview has several limitations. Firstly, as a retrospective analysis, the data curation was subject to potential biases related to data collection and missing information. Secondly, some diagnostic criteria and treatment approaches have evolved over time, which may have impacted the consistency of the data. Finally, the long-term follow-up data were limited, restricting the ability to assess outcomes over an extended period.

## 4. Conclusions

In conclusion, liver disease represents a significant clinical manifestation in a substantial proportion of CVID patients, primarily resulting from immune dysregulation, autoimmunity, and lymphocytic infiltration. Since delayed recognition significantly worsens patients’ outcomes, it is important for clinicians to be aware of this potential complication. Enhanced screening protocols and timely therapeutic interventions are essential for improving patients’ prognoses. Furthermore, additional research is required to further elucidate the pathophysiological mechanisms underlying liver disease in CVID, which may facilitate the development of more effective treatments and, potentially, preventative strategies.

## Figures and Tables

**Table 1:** Main liver manifestations of CVID.

Liver Manifestation	Pathology	Clinical Features	Diagnostic Approach
Nodular Regenerative Hyperplasia (NRH)	No fibrosis, small parenchymal nodules, impaired blood flow (microvascular thrombosis).	Hepatomegaly; Ascites; Non-cirrhotic portal hypertension (in advanced cases); Splenomegaly.	Elevated liver enzymes (AP, GGT). Imaging: Abdominal ultrasound (poor detection of small nodules), CT/MRI, liver biopsy.
Autoimmune Hepatitis (AIH)	Interface hepatitis, periportal fibrosis, bridging fibrosis in severe cases.	Elevated AST, ALT, GGT; Fatigue; Jaundice.	Immunoserology: Autoantibodies ANA, Anti-SMAs, Anti-LKM, Anti-SLA, p-ANCA. Liver biopsy: Interface hepatitis, lymphocytic infiltration. Exclude viral hepatitis and other causes of hepatitis.
Granulomatous Hepatitis	Non-caseating granulomas associated with T-cell dysregulation.	Chronic inflammation; Fatigue; Weight loss; Jaundice; Hepatomegaly; Splenomegaly.	Liver biopsy: Granulomas, often sarcoid like. Imaging: Transabdominal ultrasound, elastography, CT/MRI to detect hepatomegaly and fibrosis.
Portal Hypertension (Secondary to NRH)	Compression of portal veins by regenerative nodules, altered blood flow, increased resistance.	Splenomegaly; Neutropenia; Thrombocytopenia; Ascites; Varices (esophageal, gastric).	Imaging: Doppler ultrasound, CT, MRI for portal system evaluation. Biopsy for confirmation of underlying NRH or AIH.

Abbreviations: AP: alkaline phosphatase; GGT: gamma-glutamyl transferase: AST: aspartate transaminase; ALT: alanine trans-aminase; ANA: antinuclear antibodies; SMAs: smooth muscle antibodies; LKM: liver–kidney microsome; SLA: soluble liver antigen; p-ANCA: antineutrophil cytoplasmic antibodies with a perinuclear staining pattern; CT: computed tomography; MRI: magnetic resonance imaging.

## Data Availability

The data used for this report are available from the corresponding author upon reasonable request.

## Abbreviations

The following abbreviations are used in this manuscript:

AIH	Autoimmune hepatitis
ALT	Alanine transaminase
ANA	Antinuclear antibodies
AP	Alkaline phosphatase
AST	Aspartate transaminase
CT	Contrast-enhanced computed tomography
CVID	Common variable immunodeficiency
GGT	Gamma-glutamyl transferase
LKM	Liver–kidney microsome
MRI	Magnetic resonance imaging
NCPH	Non-cirrhotic portal hypertension
NHL	Non-Hodgkin lymphoma
NMSC	Non-melanoma skin cancer
NRH	Nodular regenerative hyperplasia
p-ANCA	Antineutrophil cytoplasmic antibodies with a perinuclear staining pattern
SLA	Soluble liver antigen
SMAs	Smooth muscle antibodies
